# Occupancy Modeling, Maximum Contig Size Probabilities and Designing Metagenomics Experiments

**DOI:** 10.1371/journal.pone.0011652

**Published:** 2010-07-29

**Authors:** Stephen A. Stanhope

**Affiliations:** Biological Sciences Division, University of Chicago, Chicago, Illinois, United States of America; Miami University, United States of America

## Abstract

Mathematical aspects of coverage and gaps in genome assembly have received substantial attention by bioinformaticians. Typical problems under consideration suppose that reads can be experimentally obtained from a single genome and that the number of reads will be set to cover a large percentage of that genome at a desired depth. In metagenomics experiments genomes from multiple species are simultaneously analyzed and obtaining large numbers of reads per genome is unlikely. We propose the probability of obtaining at least one contig of a desired minimum size from each novel genome in the pool without restriction based on depth of coverage as a metric for metagenomic experimental design. We derive an approximation to the distribution of maximum contig size for single genome assemblies using relatively few reads. This approximation is verified in simulation studies and applied to a number of different metagenomic experimental design problems, ranging in difficulty from detecting a single novel genome in a pool of known species to detecting each of a random number of novel genomes collectively sized and with abundances corresponding to given distributions in a single pool.

## Introduction

Recent experiments in metagenomics (also known as community genomics or environmental genomics) have proposed that genetic sequences from previously uncatagologued species can be discovered/recovered and investigated by subjecting large samples of RNA or DNA taken from a pool of organisms representative of a set of different species to shotgun sequencing and assembly [1]–[7]. Such a technique is regarded as especially useful for obtaining genetic information from species resistant to standard culturing techniques (e.g. [8], [9] provide recent overviews of the state of microbial cultivation; approximately 99% of microorganisms are suggested to be resistant), and is presumed to is presumed to yield contigs that are representative of the collection of species in the sample (Mavromatis *et al* [10] provides an evaluation of this and related claims).

To date, there are many specific examples of metagenomic studies. In an early project viruses isolated from seawater samples were lysed and the recovered DNA molecules were then sequenced and assembled, yielding contigs from a number of previously unsequenced virus species [11]. This was followed by a number of additional sea and ocean water analyses that investigated issues relating to microbial diversity, phylogeny, structure and function [12]–[15]. In extensions of this general program to other environments, the microbial contents of sediment samples [16], [17]; hot springs and hydrothermal vents [18]–[20]; soil [21], [22]; and other environments [23]–[25] have been similarly studied. More recently, the human metagenome is seeing attention from the metagenomics community [26]. In [27], viruses in human fecal matter were isolated and sequenced as were those in seawater. Again, this initial study was followed by others of the human gut [28]–[31] and blood [32]. It can be anticipated that further human studies will continue to be proposed and performed.

As metagenomics finds continued application, it is desirable that studies are well planned and that appropriate procedures are developed for the analysis of the data. The development of computational and statistical procedures for evaluating data collected in metagenomics experiments is ongoing [33], and is not the direct focus of this paper. Rather, we are concerned with basic properties of the assembly that can be derived from first principles and used to guide experimental protocols.

For sequencing experiments in which the genome of a single isolated organism is analyzed, a number of results relating read count to expected coverage and depth of coverage have been obtained (e.g. [34]–[36] as summarized by [37], Chapter 5.1) and adding to this body of knowledge continues to be an area of active research [38]–[43]. In the field of metagenomics, investigations into experimental design methodology have focused on extending the Lander-Waterman coverage model [35] to handle pools of species [33]. While this provides one possible metric for experimental design, it is unclear that full control over the number of reads per species is reasonable due to uncertainty regarding the number of species present in an uncontrolled sample of organisms and the degree to which genetic heterogeneity between organisms of the same species exists. In particular, if the number of species represented by organisms or genetic heterogeneity between organisms in the pool is greater than anticipated coverage and depth of coverage will be less than otherwise expected. Alternatively, if the number of reads is set to achieve a given depth of coverage on a hypothetical species with low abundance then high abundance species can be substantially oversampled [5]. Irrespective of the technical and practical issues related to extending the Lander-Waterman approach for use on metagenomics problems, it is unclear that the coverage/depth of coverage metric is an appropriate one for all experiments. In particular, for experiments designed to assess numbers of species represented in a sample or discover the presence of novel species in a sample containing primarily organisms from known species, it may be deemed unnecessary to achieve a high degree of coverage. Instead, simply discovering contigs of appropriate size representative of individual species in the sample or obtaining a single reasonably sized contig from a novel species may be desired.

In this paper, we propose that the probability of obtaining at least one contig of a minimum specified size without restriction based on depth of coverage from the genomic assembly of reads corresponding to a given novel species provides a metric representative of a desirable outcome for metagenome sequencing studies in which relatively small numbers of reads per species can be anticipated. We obtain an approximate measure of this probability for single genome studies, and present four applications of it to hypothetical metagenome sequencing studies of increasing difficulty. In the first, we design an experiment in which the goal is to obtain a contig of a given minimal size from a single novel species of specified genome length that is represented by organisms pooled in equal proportion with those from a large number of known species of identical genome size. In the second we design an experiment in which the goal is to obtain appropriately sized contigs simultaneously from each of a large (but specified) number of novel species of equal genome size and representation in a pool of organisms containing no known species. We extend this result to experiments in which genome sizes and abundances vary across species, and then further to allow the pool size to be regarded as random and genome sizes and abundances to be collectively distributed according to specified measures. We verify both our approximation of the distribution of maximum contig sizes for the assembly of a single genome and experimental designs for random pools and distributed genome sizes/abundances by simulation.

## Results

### Largest contig size probabilities for a single genome

**Figure 1:**
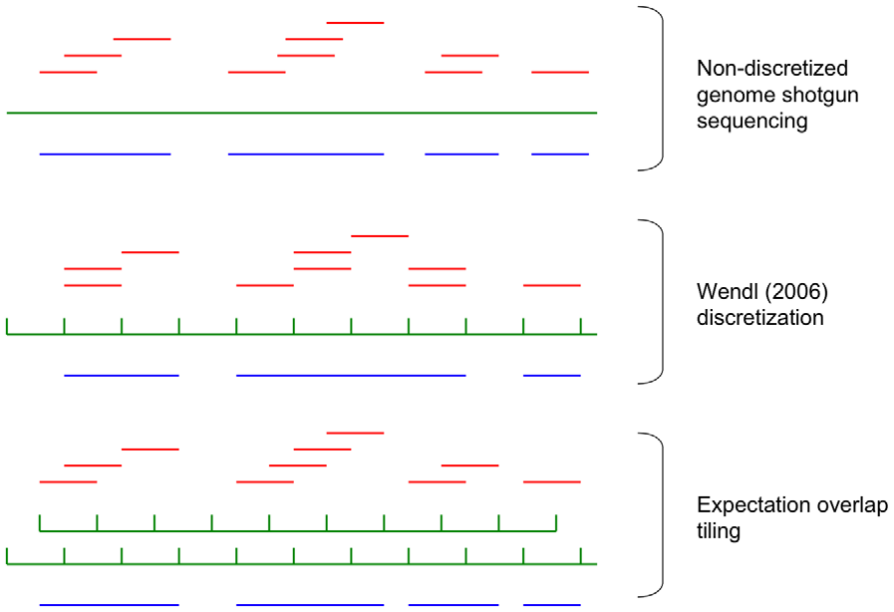
Discretizations of shotgun genome sequencing. In the non-discretized model, reads (red) are derived from a genome (green) and assembled into contigs (blue). Contig assembly relies on overlap between reads. In the Wendl (2006b) discretization, the genome is partitioned into a number of read-sized bins. Reads are distributed amongst these bins, and a contig can be regarded as a sequence of occupied bins. In the expectation overlap tiling, a secondary set of read-sized bins overlap those from the Wendl discretization, and a contig of size defined in an integer number of bins can be obtained from a sequence of occupied Wendl or overlap bins independently.

Let 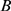 be the length of a candidate genome, and let 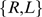 be the anticipated number of reads of that genome and length of an individual read. The probability of obtaining at least one contig of a minimum specified size *k* from the assembly of those reads is equal to the probability that the longest contig is at least size *k*, and letting 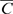 be the size of the longest contig in the assembly 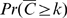 is to be assessed. To do so, we utilize recent results by Wendl [41] that model coverage by discretizing the genetic sequence into 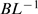 read-sized bins (Fig. 1) and assuming reads to be equally distributed amongst those bins. This approximation was originally used to obtain a measure of coverage probability, which provides an alternative sequencer experiment design paradigm from the expectation-based metrics more typically considered (e.g. [35]).

**Figure 2:**
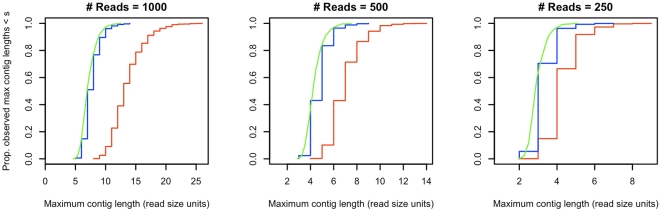
Simulated maximum contig size distributions. Fig. 2 presents sample cumulative distribution functions of maximum contig sizes obtained through simulations of contigs assembled from 1000, 500 and 250 reads of length 200 on a hypothetical genome of 200000 bases. The green, red and blue lines represent samples from the non-discretized genome, Wendl-discretized genome, and expectation overlap tiled genome respectively. The Wendl discretization yields substantial overestimates of the probability of obtaining contigs of at least a desired size. The expectation overlap tiling yields an improved approximation.

To determine whether direct use of the occupancy approximation can be used to obtain maximum contig size probabilities, we compared simulated distributions of maximum contig sizes from reads assembled on a hypothetical genome before and after discretization. A single iteration of the simulation of a non-discretized genome operated by defining an array of bases, accumulating reads of a defined length onto that array, and computing the size of the largest contiguous region of occupied bases. The sample cumulative distribution function of the largest observed contiguously occupied region sizes over all iterations was then plotted. The simulation of the discretized genome operated analogously, with an array of 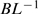 bins and an accumulation of reads of length 1 into those bins. Fig. 2 compares the resulting distribution functions of maximum contig size (measured in read lengths) from the non-discretized (green) and Wendl discretization (red) genome simulations for the case 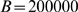, 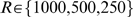, 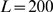 (this selection of genome size corresponds to that of a generic virus, and the read length approximates that from a 454 pyrosequencer) over 1000 iterations. It is apparent that the discretization proposed by Wendl substantially overestimates 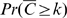.

To obtain an improved approximation to the actual distribution of longest contig sizes, we propose an alternative discretization of the genetic sequence into 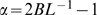 bins, oriented such that each neighboring pair of the 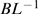 bins obtained using the Wendl distribution are overlapped by an additional bin (Fig. 1). This discretization is motivated by the principle that the average overlap between two reads that form a single contig is sized at half the length of a read, and so we refer to it as “expectation overlap tiling.” After expectation overlap tiling, contigs of size 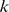 in read-lengths are obtained by achieving a 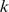-long sequence of neighboring bins in either the original Wendl discretization or the overlap bins. (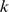-long contigs formed by alternating between a total of 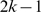 Wendl and overlap bins are a subset of 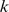-long sequences of bins in either the Wendl or overlap bins, and need not be considered.) To determine whether this procedure yielded maximum contig size distributions with better fidelity to those obtained in the nondiscretized case, we conducted a simulation in the same manner as those previouly described. Fig. 2 provides the cumulative distribution of maximum contig sizes from the expectation overlap tiled genome in blue. It is clear that it reasonably approximates that of the non-discretized case.

A formal expression for 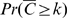 can be obtained using the expectation overlap tiling by assuming that reads are equally distributed amongst bins and then deriving appropriate occupancy and run length probabilities, as in [41]. We suppose that each read is mapped to bin 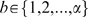 with probability 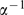, and that the probability that bin *b* contains contains at least one read is 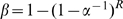. Let 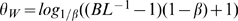 and 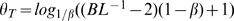. Then:
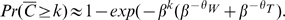
(1)can be derived as described in Methods.

**Figure 3:**
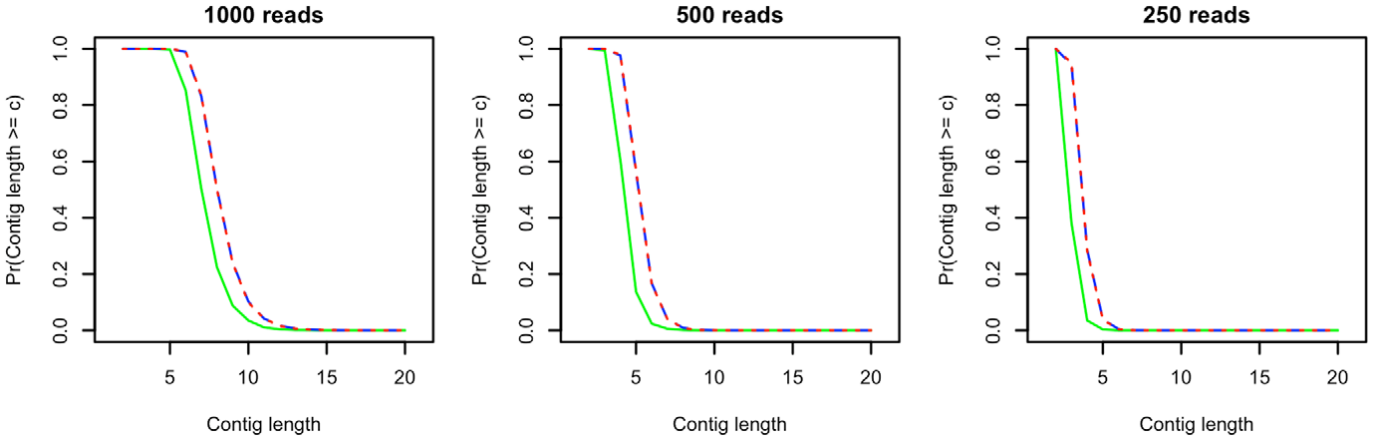
Maximum contig size probabilities, virus sequencing. Fig. 3 provides estimated and analytically determined probabilities of maximum contig sizes for genomes of 200000 bases sequenced using 1000, 500 and 250 reads of length 200. The green, red and blue lines represent probabilities determined using simulations of the non-discretized and expectation overlap tiled genomes, and Eq. 1 respectively. Eq. 1 accurately represents maximum contig size probabilities determined from the expectation overlap tiled genome, and slightly overestimates true probabilities as determined by the non-discretized model.

**Figure 4:**
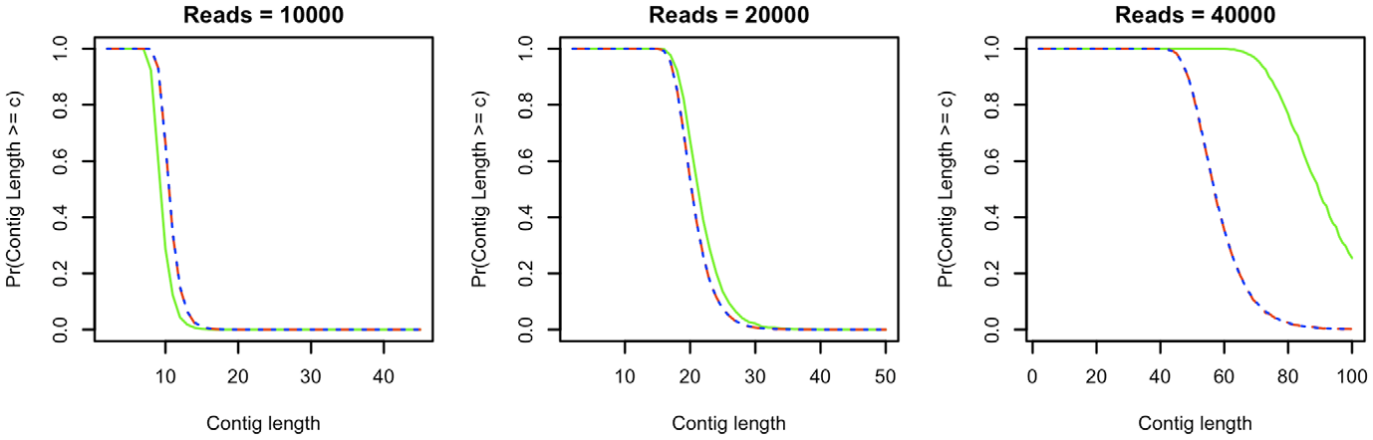
Maximum contig size probabilities, bacterium sequencing. Fig. 4 provides estimated and analytically determined probabilities of maximum contig sizes for genomes of 2000000 bases sequenced using 10000, 20000 and 40000 reads of length 200. The green, red and blue lines represent probabilities determined using simulations of the non-discretized and expectation overlap tiled genomes, and Eq. 1 respectively. For relatively low coverage levels Eq. 1 accurately estimates actual maximum contig size probabilities as determined by simulations of the non-discretized genome. However, it is inaccurate when the number of reads is 40000, corresponding to a 4× depth of coverage.

To demonstrate the accuracy of Eq. 1, we compared longest contig size probabilities determined analytically to those obtained through simulations similar to those used in Fig. 2. In these simulations, maximum contig sizes were estimated from 10000 simulated assemblies of the non-discretized genome (the desired standard) and the expectation overlap tiled genome. Fig. 3 provides the results of this analysis for the previously studied virus sequencing problem (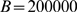, 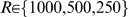, 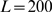). Additionally, we consider a problem analogous to sequencing a bacterium at a higher level of coverage than the virus problem (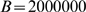, 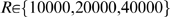, 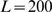) in order to study the performance of the model for both larger genomes and greater coverage levels. The results of this analysis are provided in Fig. 4.

In Fig. 3, simulation-based maximum contig size probabilities from the non-discretized and expectation overlap tiling discretized genomes are in green and blue dashed lines respectively. and red dashed lines represent analytically determined probabilities. We note that Eq. 1 accurately represents maximum contig size probabilities obtained from the simulation of the expectation overlap tiled genome, demonstrating that the analytical model operates as anticipated. Consistent with this and what was observed in Figure 2, maximum contig size probabilities from either the expectation tiled genome simulation or Eq. 1 slightly overestimate the true probabilities. More detailed investigation suggests that the size of overestimation is approximately one contig (i.e. the probability of obtaining a contig of at least length 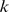 as determined by Eq. 1 is approximately equal to the probability of obtaining a contig of at least length 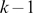 in the non-discretized genome simulation). Fig. 4 yields similar results for the cases in which low numbers of reads (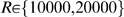) are utilized, although for 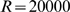 Eq. 1 actually slightly underestimates maximum contig sizes. The underestimation of maximum contig sizes becomes extreme when 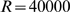, which represents a 4× coverage level of the genome and suggests a technical limitation of the model to those cases in which a relatively small number of reads per genome are available. Because metagenomics sequencing studies are typically anticipated to yield a relatively small number of reads per individual genome or species, Eq. 1 is appropriate for use in approximating the distribution of maximum contig sizes in such problems.

### Detecting a single novel species in a pool of known species

To design an experiment in which the goal is to obtain a contig of at least a given size from a single novel species that is pooled with a large number of known species, we suppose that there are 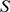 known species in the pool, all species in the pool (including the novel one) are of length 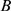, and that reads are of length 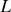. For convenience, we assume that all species have the same relative abundance and for a given number of total reads 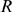 marginally 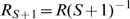 reads are expected to be allocated to the novel species. 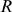 is to be set such that a contig of at least size 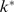 from the novel species will be observed with probability 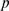. As described in Methods, 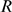 will meet this design goal if it is such that:
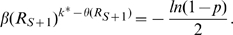
(2)where 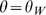, and 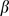 is expressed as a function of read count.

**Figure 5:**
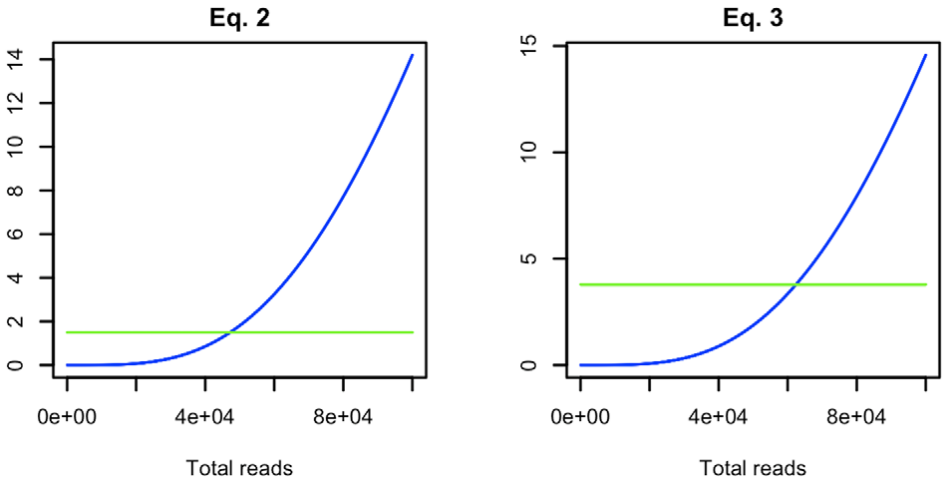
Experimental designs for detecting a single species and obtaining contigs representative of a pool of genomes. Intersection between the left (blue) and right (green) sides of Eqs. 2 and 3 indicate the number of length 200 reads necessary to have 95% confidence of obtaining at least one contig with minimal size of 4 reads from a novel genome of length 200000 bases pooled with 100 like-sized genomes, and from each of 100 pooled genomes of length 200000 respectively. Detecting a single novel species requires 47213 reads, expected to allocate 467 to the novel species. Detecting contigs representative of the pool of genomes requires 62402 reads, expected to allocate 624 to each species. These results are consistent with those described in Figs. 3 and 4.

Although Eq. 2 does not offer a closed form solution, an algorithm for obtaining 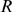 such that the equality is met can be implemented in a straightforward manner. To demonstrate this, we consider a multiple virus sequencing problem in which 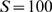 species of length 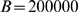 are to be sequenced with reads of length 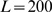. Suppose that there is a single additional novel virus in the pool for which a contig of at least length 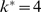 is to be observed with probability 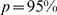. This problem corresponds to those analyzed previously, and as demonstrated in Fig. 3 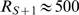 and a total number of experimental reads 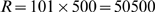 is expected to be needed to achieve this goal. Fig. 5 provides the relationship of the left and right sides of Eq. 2 (blue and green lines respectively) as a function of 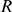. Equality is obtained at 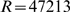, and 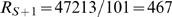.

### Obtaining contigs representative of a pool of species

We continue our application of Eq. 1 by using it to design an experiment in which the goal is to obtain an appropriately sized contig from each of a large number of novel species simultaneously. We suppose that there are 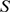 novel species of equal commonality and length 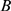 in the pool, and that reads are of length 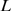. For a given number of total reads 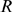 marginally 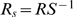 reads are expected to be allocated to each. 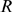 is to be set such that a contig of at least size 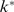 will be obtained from each species with with probability 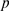. Based on this, the condition analogous to Eq. 2 is:
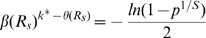
(3)(see Methods for derivations).

As previously, an algorithm for obtaining 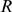 such that the equality in Eq. 3 is met was implemented and tested on a virus sequencing problem with 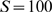 species of length 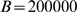, sequenced with reads of length 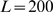. The design problem is to calculate the total number of reads 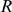 such that contigs of at least length 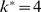 would be obtained from each species in the pool with with probability 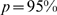. Again, this problem corresponds roughly with those previously examined, although the requirement that appropriately sized contigs were to be obtained from each species in the pool rather than a single species is anticipated to increase the number of reads per species necessary. Fig. 5 provides the relationship of the left and right sides of Eq. 3 as a function of 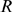. Equality is obtained at 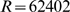 and 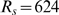 per species, approximately a 30% increase from that required to achieve the same performance from our single species problem. This increase in the required number of reads can be attributed to the necessity of obtaining a contig of the desired size from each species in the sample, rather than only a single novel species. To further study the behavior of experimental designs using Eq. 3, we calculated designs for a number of different hypothetical viral and bacterial metagenome experiments. The results of our calculations as a function of 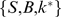 are provided in Tables 1 and 2. We note that as might be reasonably anticipated, increases in 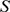, 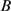 and 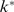 all yield increases in the number of reads necessary to obtain contigs of the desired size from each species in the pool with 95% probability.

**Table 1:** Designs for viral metagenome experiments.

S ( 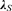 )	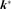	Eq. 3 Reads	Eq. 4 Reads	Eq. 5 Reads	(5,50,95)% minimax
100	4	62402	62166	67109	3.38, 3.68, 3.94
200	4	128399	135996	142673	3.32, 3.60, 3.80
400	4	263645	303155	310081	3.24, 3.49, 3.72
100	5	88636	89303	96992	4.43, 4.85, 5.15
200	5	181745	196402	206985	4.28, 4.70, 5.04
400	5	371999	438059	449314	4.23, 4.56, 4.78
100	6	113767	115738	126271	5.63, 6.16, 6.50
200	6	232749	255203	269879	5.39, 5.92, 6.27
400	6	475413	569204	585113	5.17, 5.73, 6.04

Table 1 provides the numbers of reads of size 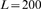 determined to give 95% probability of assembling contigs of at least size 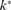 in viral (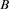 = 200000, 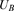 = Uniform(50000,350000)) metagenomics problems as a function of the number of species 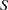 or 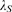 in the pool. Calculations are provided for models using fixed pool and equal genome sizes and abundances (Eq. 3), fixed pool sizes with distributed genome sizes and abundances (Eq. 4) and stochastic pool sizes with distributed genome sizes and abundances (Eq. 5). (5, 50, 95)% minimax contig size quantiles from simulated assemblies of 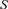 species with uniformly distributed genome sizes and Pareto distributed abundances using stochastic pool size/distributed genome size and abundance experimental designs are provided for verification. Larger numbers of reads are required to obtain a given level of performance as pool sizes increase, the required performance level increases, if an assumption of equal genome sizes and abundances is replaced with one of distributed genome sizes/abundances with equivalent mean genome sizes, or if a fixed pool size is replaced with a stochastic pool. Consistent with previous observations, minimax contig size quantiles are slightly (less than one read length) lower than planned.

**Table 2:** Designs for bacterial metagenome experiments.

S ( 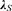 )	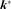	Eq. 3 Reads	Eq. 4 Reads	Eq. 5 Reads	(5,50,95)% minimax
100	4	313476	336963	365122	3.33, 3.49, 3.65
200	4	642683	766366	807394	3.27, 3.44, 3.59
400	4	1315088	1764672	1806689	3.21, 3.38, 3.51
100	5	489834	535031	584071	4.32, 4.51, 4.75
200	5	1000506	1217621	1290028	4.17, 4.43, 4.61
400	5	2040273	2800352	2877594	4.13, 4.37, 4.59
100	6	669257	739677	811931	5.26, 5.63, 5.92
200	6	1363646	1683594	1791344	5.20, 5.57, 5.83
400	6	2774570	3868329	3986651	5.06, 5.36, 5.59

Table 2 provides the numbers of reads of size 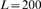 determined to give 95% probability of assembling contigs of at least size 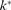 in bacterial (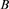 = 2000000, 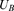 = Uniform(1000000,3000000)) metagenomics problems as a function of the number of species 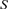 or 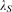 in the pool. Calculations and relationships between both experimental terms and the required number of reads and planned and observed contig sizes are as described in Table 1.

### Fixed pool sizes with distributed genome sizes and abundances

A general extension of the result described in Eq. 3 to problems with varying genome sizes and abundances can be obtained, although it does not result in an easily managed experimental design criterion such as in Eq. 3. For species 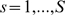 let 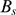 be the genome size and 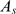 the percentage abundance (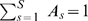). Let 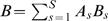 be the abundance normalized total genome size. The criteria to be met for obtaining contigs of at least size 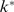 from all species with probability 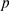 is:
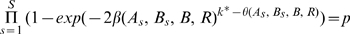
(4)where 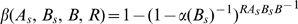, 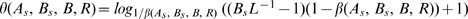, 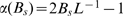, and the number of reads allocated to each species is now dependent on its proportional representation in the total genome (see Methods).

To use Eq. 4 to derive experimental designs, individual genome sizes and abundances must be specified. Treating these quantities as random variables would lead to an intractable integral, and therefore we choose to collectively set them such that desired aggregate genome size and abundance distributions are met across the pool. We begin by noting that substantial variability in genome size distributions has been observed in previous metagenomic studies (e.g. [44], [45]). In order to avoid issues with the shape of the selected distribution, we suppose that genome sizes are to be collectively uniformly distributed and we let 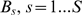 be the 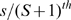 quantiles of a Uniform(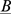, 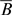) distribution. Next, we let 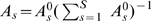 where 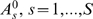 are species abundances normalized to the commonality of the least abundant species. We suppose that 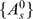 are Pareto-distributed with scale and shape parameters 1 and 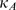 respectively, and we let 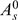 be the 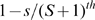 quantiles of that distribution. We note that this assignment of abundances of species models a case in which large genomes are relatively rare compared to small genomes, and letting 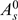 be the 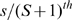 quantile would model the opposite. In our implementation of a solver for Eq. 4, selection of either abundant large or small genomes is provided as an option. Derivations performed in Methods are for general genome size and abundance distributions, and changes in such assumptions can be made without a substantial change in our methodology.

After designing an algorithm for obtaining 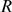 such that the equality in Eq. 4 is met, we tested it on a virus sequencing problem with 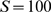 species and lengths uniformly distributed with between 50000 and 350000 bases. This corresponds to virus genome size bounds described by [46] and is such that the mean size was 200000, consistent with what was previously examined. Abundances were supposed to be Pareto(1,3.5). The selection of scale parameter for the abundance distribution was made such that the most abundance species represented 2.72% of the sample, which is consistent with previous metagenomic analyses [11]. Contigs of at least length 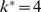 were to be obtained from each species in the pool with with probability 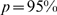 using reads of length 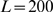. This problem specification yielded a design with 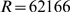, a 0.4% decrease from the number of reads required for the same performance if genome lengths were held constant with 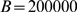 and abundances were assumed to be equal. (The fact that the two designs are equal can be likely attributed to the inverse relationship between abundance and genome size, combined with the small size of the population.) We additionally computed equivalent designs for the experiments described in Tables 1 and 2. In these experiments, as the number of species was increased 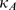 was reset so as to maintain an approximate 2.5% representation of the most abundant species. (We continued to use a Uniform(50000,350000) distribution of genome sizes.) For the cases considered, assuming distributed genome lengths and abundances yielded designs that used 99.6%–139% of the number of reads than those computed for experimental designs assuming constant genome lengths and abundances. The differences between the two designs increase for larger pool sizes, 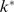 and genome sizes.

### Stochastic pools with distributed genome sizes and abundances

We conclude our applications of our model of maximum contig size probabilities by extending our previous results to weaken the requirement of specifying a fixed pool size, in order to more realistically represent the uncertainties in actual metagenomics experiments. We do so by modeling pool size as a random variable. Let 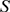 be distributed Poisson with mean 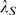. We suppose that conditional on 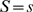, genome sizes and commonality normalized abundances will be uniformly and Pareto-distributed as previously, with 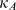 a function of 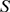 such that the maximum percentage abundance is equal to 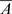. The total number of reads 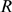 such that a contig of at least size 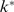 will be obtained from each of the random number of species with with probability 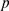 meets the following condition:
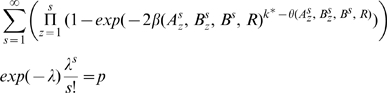
(5)where the dependence of individual abundances and genome sizes on 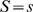 is made explicit; such dependence is due to the use of quantiles of distributions to set these values. See Methods for derivations using general distributions of 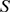.

Building on previous analyses, we designed and tested an algorithm for obtaining 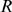 such that the equality in Eq. 5 is met on a virus sequencing problem with 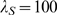 species with lengths uniformly distributed with between 50000 and 350000 bases and abundances meeting a Pareto distribution such that the maximum abundance is 2.5%, ordered such that smaller genomes have greater abundance. Contigs of at least length 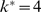 were to be obtained from each species in the pool with with probability 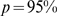 using reads of length 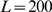. This problem specification yielded a design with 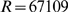, an 8% increase from the number of reads required for the same performance if pool size was held constant at 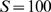. We computed equivalent designs for the experiments described in Tables 1 and 2, again using Poisson-distributed pool sizes, uniformly distributed genome sizes and Pareto distributed abundances such that the most prevalent genome represented 2.5% of the total sample. For the cases considered here, this yielded a 2%–10% increase in the number of reads than those computed for experimental designs assuming fixed pool sizes and distributed genome lengths and abundances.

**Figure 6:**
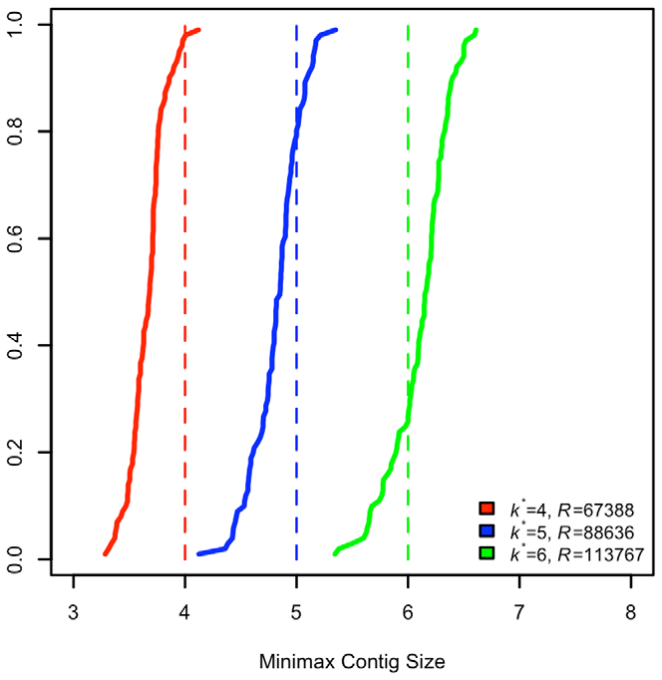
Minimax contig sizes observed for simulated viral metagenome assemblies. For a viral metagenome experiment design based on a Poisson number of species, uniformly distributed genome sizes and Pareto distributed abundances (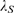 = 100, 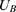 = Uniform(50000,350000), 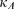 = 3.5), 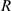 = 67109, 96992 and 126271 were calculated to have 95% probability of yielding assembled contigs of at least size 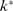 = 4, 5 and 6 for all species respectively. In Fig. 6, we show the distribution of minimax contig sizes obtained from 100 simulations of an assembly of these numbers of reads on a pool of 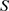 = 100 species with Uniform(50000,350000)-distributed genome sizes and Pareto(1,3.5)-distributed abundances (solid lines) vs. their targeted sizes (dashed). Consistent with previous observations for this case, the actual contig sizes obtained are slightly smaller than the targeted length. The median minimax contig sizes are 3.68, 4.85 and 6.16 (in read lengths, which is 92–103% of the target length), and 95% of all experiments yield contigs of length 3.38, 4.43 and 5.63 from all species (85–94% of the target length). The slight undersizing of contigs is consistent with previous observations (e.g. Figs. 3 and 4).

To determine whether experimental designs obtained from Eq. 5 could be expected to perform appropriately, a final simulation experiment was performed. For each of the experimental designs described in Tables 1 and 2, we performed 100 simulated assemblies of the number of reads suggested by the stochastic pool and distributed genome size and abundance model on the expected number of species used to calculated the design with genome sizes and abundances distributed according to the assumed model. In each simulated assembly, individual reads were randomly assigned to species and accumulated onto genomes in the manner described for simulations of non-discretized genome assemblies. After all reads were assigned, maximum contig sizes were computed for each genome, normalized to read lengths, and the minimum of these (referred to here as the minimax contig size) was recorded. The minimax contig size corresponds to the targeted contig size used in the experimental design, and therefore was anticipated to be approximately equal to 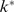 with a bias towards being slightly smaller, consistent with what was observed in Figs. 3 and 4. Tables 1 and 2 provide 5%, 50% and 95% quantiles of the observed minimax contig sizes, and Fig. 6 plots the distributions of minimax contig sizes for three viral metagenome designs. As anticipated, minimax contig sizes were typically under the designed contig length, but only slightly (less than one read length) so.

## Discussion

In metagenomics experiments, large samples of genomic material from organisms representing a number of different species are simultaneously sequenced and assembled. Although such analyses have some similarities to more typical sequencing experiments in which a single genome is studied in isolation, the change in problem context justifies an evaluation of the body of analytical and computational technique that has been developed for single organism problems, and where appropriate the development of new tools. Currently, some research effort is being put towards developing such tools for the analysis of sequence data after is has been collected. This paper is concerned with analytical technique that can be used to plan such collections.

For single genome sequencing studies, Lander and Waterman [35] have provided useful experimental design metrics based on expected coverage and depth of coverage that continue to be in use today, and in the metagenomics community some attention has been paid to appropriately extending their results. However, the metrics of expected coverage and depth of coverage may be argued to not be appropriate for all metagenomics experiments. Rather, for some experiments it may be desirable to obtain results that relate experimental protocols to the probability of obtaining a given level of coverage (as obtained by Wendl for single genome studies) or a contig of at least a given size from a particular species, as studied here. Such probability-based metrics may be used on their own, or combined with expectation-based metrics to obtain a fuller perspective of the relationship between the planned number and size of reads and the results of a proposed sequencer-based metagenomics experiment.

In this paper, we showed that the probability of obtaining a contig of a minimum specified size from an assembly of a relatively small number of reads from a single genome can be obtained by discretizing the genome into read sized bins in a modified version of the discretization suggested by Wendl [41], and then applying Poisson approximation. We verified the accuracy of this calculation in several simulation studies, and we used it to solve a number of experimental design problems representative of those addressed in metagenomics experiments. In increasing order of difficulty, we considered: 1) the design of an experiment in which the goal is to discover evidence of a novel species that is pooled with a large number of organisms from known species; 2) the design of an experiment that is intended to ascertain the number of species in a pool of equally sized and abundant previously unobserved species; 3) an extension of (2) in which genome sizes and abundances were collectively uniformly and Pareto-distributed; 4) an extension of (3) in which the pool size was regarded as random and Poisson-distributed. The derivations leading to (3) and (4) were performed for general distributions of pool and genome sizes, such that experimental designs could be obtained for other cases than examined here. As anticipated, the number of reads required to obtain a given level of performance generally increased with problem difficulty.

Presently we are investigating the extension of these results to further experimental designs as well as their utility for data analysis. All codes used in the process of writing this paper are publicly available. They are written in the R programming language and are provided in File S1 of the paper, at http://www.bioinformatics.org/maxcontigprob or by contacting the author.

## Methods

### Largest contig size probabilities for a single genome

Let 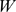 and 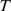 be binary random vectors representative of bin occupancy for the Wendl and overlap discretization bins respectively, and 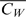 and 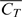 random variables representing the size of the largest run of occupied bins in 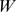 and 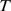. We note that:
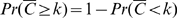
(6)

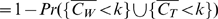
(7)

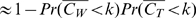
(8)where the approximation is due to dependence between 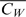 and 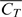 not modeled here. To obtain the distribution of 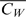 and 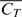 we utilize the application of Poisson approximation to the calculation of runs in sequences of Binomial random variables by [47] as described in [48] Section 4.2. Let 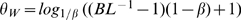 and 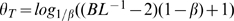. Then 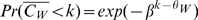, 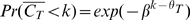 and: 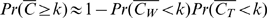
(9)

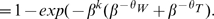
(10)


### Detecting a single novel species in a pool of known species

Practially, 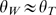 and Eq. 1 can therefore be simplifed: 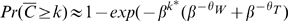
(11)

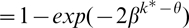
(12)where 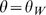. Then the condition that is to be met is:
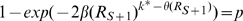
(13)where 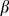 is expressed as a function of read count. Simplifying, the condition in Eq. 13 is met if:
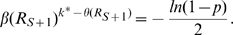
(14)


### Obtaining contigs representative of a pool of species

Conditional on 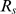 the probabilities of reads corresponding to each species assembling into a contig of at least length 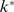 are independent across species:
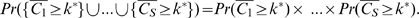
(15)Based on this, the condition analogous to that provided in Eq. 13 is:
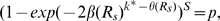
(16)which simplifies into:
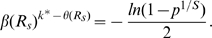
(17)


### Non-constant genome sizes and abundances

For species 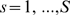 let 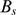 be the genome size and 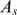 the percentage abundance (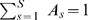). (Other measures of abundance can be used and transformed to percentage abundance.) Let 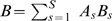 be the abundance normalized total genome size. Working from Eq. 15: 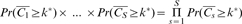
(18)

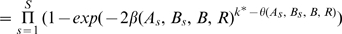
(19)where 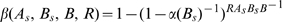, 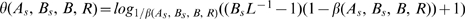, 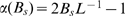, and the number of reads allocated to each species is now dependent on its proportional representation in the total genome.

To assign abundances and genome size to meet marginal distributional specifications, suppose 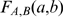 to be their joint distribution (where 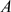 and 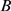 are now random variables). 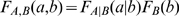, where 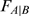 is the distribution of abundance conditional on genome size and 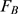 is the marginal distribution of genome sizes. For species 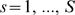, assigning 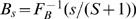 is sufficient for the sample to meet the genome size distribution requirement. If a conditional distribution of abundance conditional on genome size is proposed, then one reasonable approach would be to assign 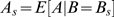. However, we consider the case in which assigned abundances are to be marginally distributed 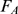, with no information regarding the dependence of 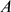 on 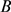. We note that any assignment of the 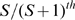 quantiles of 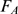 to species in the pool will meet the desired criterion. Two natural assignments to consider are 1) greater abundances to smaller genomes (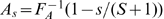) and 2) greater abundances to larger genomes (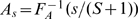). We utilize the former in this paper's examples, although the latter is provided as an option in our software implementation.

We conclude by noting that the use of differing abundances across species can result in substantial variability of coverages across organisms. Depending on the particular assignments of 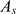 and 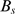, this can lead to cases in which contig size probabilities are substantially underestimated for species with high coverages (e.g. Fig. 4), which can lead to difficulties in determining a design for a desired experiment. To resolve this, in our codes for solving Eq. 4 we assign species with high coverage measures a 100% probability of obtaining a contig of the specified size. This is likely to be reasonable, considering the particulars of the metagenomics experimental design problem considered here.

### Stochastic pools, distributed genome sizes and abundances

To extend the results in Eqs. 15–19 to a pool of species that is regarded as random but characterized by a distribution, we condition on 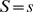 and integrate Eq. 19: 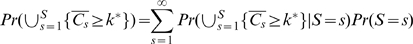
(20)

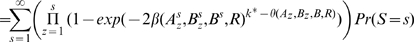
(21)where genome sizes and abundances are as previous, but now conditional on 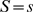 due to the use of quantile assignment. Eq. 21 is the basis for the experimental design condition provided in Eq. 5 for Poisson distributions of 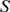.

## Supporting Information

File S1 R scripts for performing computations described in Stanhope (2010).
(0.00 MB GZ)
